# Comparison of segmentation performance of cnns, vision transformers, and hybrid networks for paranasal sinuses with sinusitis on CT images

**DOI:** 10.1038/s41598-025-17266-w

**Published:** 2025-09-01

**Authors:** Dahyun Song, Su Yang, Ji Yong Han, Kwang Gi Kim, Seon Tae Kim, Won-Jin Yi

**Affiliations:** 1 Interdisciplinary Program in Bioengineering, Graduate School of Engineering, Seoul National University, Seoul, Korea; 2 Department of Applied Bioengineering, Graduate School of Convergence Science and Technology, Seoul National University, Seoul, Korea; 3 Department of Biomedical Engineering, College of IT Convergence, Gachon University, Seongnam, Korea; 4 Department of Otolaryngology-Head and Neck Surgery, Gachon University Gil Hospital, Incheon, 21565 Korea; 5 Department of Oral and Maxillofacial Radiology and Dental Research Institute, School of Dentistry, Seoul National University, Seoul, 03080 Korea

**Keywords:** Paranasal sinus, Sinusitis, 3D image segmentation, Convolutional neural network (CNN), Vision transformer (ViT), Hybrid network, Biomedical engineering, Computed tomography, Diseases, Machine learning

## Abstract

Accurate segmentation of the paranasal sinuses, including the frontal sinus (FS), ethmoid sinus (ES), sphenoid sinus (SS), and maxillary sinus (MS), plays an important role in supporting image-guided surgery (IGS) for sinusitis, facilitating safer intraoperative navigation by identifying anatomical variations and delineating surgical landmarks on CT imaging. To the best of our knowledge, no comparative studies of convolutional neural networks (CNNs), vision transformers (ViTs), and hybrid networks for segmenting each paranasal sinus in patients with sinusitis have been conducted. Therefore, the objective of this study was to compare the segmentation performance of CNNs, ViTs, and hybrid networks for individual paranasal sinuses with varying degrees of anatomical complexity and morphological and textural variations caused by sinusitis on CT images. The performance of CNNs, ViTs, and hybrid networks was compared using Jaccard Index (JI), Dice similarity coefficient (DSC), precision (PR), recall (RC), and 95% Hausdorff Distance (HD95) for segmentation accuracy metrics and the number of parameters (Params) and inference time (IT) for computational efficiency. The Swin UNETR hybrid network outperformed the other networks, achieving the highest segmentation scores, with a JI of 0.719, a DSC of 0.830, a PR of 0.935, and a RC of 0.758, and the lowest HD95 value of 10.529 with the smallest number of the model architectural parameter, with 15.705 M Params. Also, CoTr, another hybrid network, demonstrated superior segmentation performance compared to CNNs and ViTs, and achieved the fastest inference time with 0.149 IT. Compared with CNNs and ViTs, hybrid networks significantly reduced false positives and enabled more precise boundary delineation, effectively capturing anatomical relationships among the sinuses and surrounding structures. This resulted in the lowest segmentation errors near critical surgical landmarks. In conclusion, hybrid networks may provide a more balanced trade-off between segmentation accuracy and computational efficiency, with potential applicability in clinical decision support systems for sinusitis.

## Introduction

The paranasal sinuses, comprising the frontal sinus (FS), ethmoid sinus (ES), sphenoid sinus (SS), and maxillary sinus (MS), play a central role in thermoregulation during rapid temperature fluctuations, facilitate ventilation and drainage, and provide structural protection against facial trauma^1,2^. Sinusitis refers to inflammation of the mucosal lining of the paranasal sinuses and is commonly classified as either acute or chronic based on duration and clinical characteristics^2^. Chronic rhinosinusitis (CRS), in particular, is defined as a persistent inflammatory condition lasting longer than 12 weeks, and is primarily characterized by mucosal thickening and impaired sinus drainage^2^. CRS can lead to epithelial remodeling and periosteal inflammation, particularly affecting the ethmoid bone^3^. Although CRS may present with mucopurulent rhinorrhea in some cases, this finding is more characteristic of acute sinusitis. In contrast, CRS typically involves nasal congestion, facial pressure, and postnasal drip. Prolonged inflammation can result in mucosal hypertrophy and bony remodeling^3^. Because the paranasal sinuses are anatomically close to critical structures such as the orbit and cranial nerves, accurate assessment of sinus inflammation is clinically important, particularly in chronic cases where mucosal thickening and anatomical variations may complicate treatment planning^1–3^.

CT scans are an essential tool for diagnosing sinusitis, as they can detect inflammation in the sinus cavities. After identifying inflammation, clinicians consider a combination of imaging findings, symptom severity, and anatomical extent to determine the appropriate medical or surgical treatment^4,5^. Segmentation of sinus structures on CT images can provide valuable support during surgical planning by helping identify anatomical variations and guide safer, more efficient navigation, particularly when used in conjunction with image guidance systems^6,7^. Importantly, preoperative segmentation has been shown to improve surgical efficiency by reducing the time required to identify critical anatomical landmarks during navigation-assisted procedures, thereby facilitating faster and safer intraoperative decision-making^7^. In addition to improving surgical workflow efficiency, the clinical applicability of segmentation tools may also depend on technical considerations such as computational efficiency, memory requirements, inference latency^7^ and the technical feasibility of deploying models in clinical environments^8^. In addition to surgical planning, automated segmentation can also support 3D volumetric staging of chronic rhinosinusitis (CRS), which quantitatively assesses the extent of sinus opacification and helps evaluate the effectiveness of therapeutic interventions^9^. This volumetric assessment, based on the percentage of disease involvement across paranasal sinuses, has been used for over a decade and is gaining attention as a more sensitive and reproducible method compared to traditional scoring systems^10^, while subsequent studies have introduced 3D image-based staging frameworks to enhance its clinical utility^11^.

Among various surgical approaches, Endoscopic sinus surgery (ESS) is a common treatment method for sinusitis. Accurate visualization of the patient’s internal anatomy is essential for surgeons during ESS^12^. To better visualize the surgical target and anatomical structures, image-guided surgery (IGS) has become a popular visual aid^13^. IGS provides continuous and enhanced visualization of anatomical structures using three-dimensional (3D) virtual structures fused with endoscopic images^12–14^. In ESS based on an IGS system, the accuracy of the surgery depends largely on the registration between the patient’s anatomical structures and the endoscopic image^13,15^. Consequently, automatic segmentation can enhance pre-operative planning, and when used in conjunction with IGS can provide improvements in the execution of the sinus surgery by providing accurate anatomical registration and detailed 3D representations of patient-specific structures^15^.

As the popularity of deep learning has grown in the field of medical imaging, several studies of automatic segmentation of the paranasal sinuses using deep learning have been reported^16–20^. The paranasal sinus regions, which are closely arranged within the skull, add complexity to segmentation tasks due to their intricate spatial configuration. The adjacent nasal cavity, cranial nerves, and optic nerve are close to these sinuses, contributing to their functional and anatomical complexity^2^. Given these characteristics, Kuo et al. segmented the ES into anterior and posterior sections for more precise analysis using CNNs^16^. Iwamoto et al. achieved refined segmentation outcomes for each sinus area by combining a fully convolution network (FCN) with a probability atlas to refine the FCN’s outputs^17^. Subsequent studies have extended such approaches into 3D volumetric staging frameworks. Kuo et al. employed semi-supervised CNNs with pseudo-label self-training for volumetric segmentation and scoring^18^, while Massey et al. demonstrated strong correlations between automated CT metrics and established clinical scores^19^. Most recently, Whangbo et al. compared the multi-class segmentation performance of several U-Net architectures, including 3D U-Net, Residual 3D U-Net, Dense 3D U-Net, and Residual-Dense 3D U-Net on CT imagery^20^. In medical imaging domains, further architectural advancements of U-Net have been proposed, such as the integration of depthwise convolution and residual connections^21^, squeeze-and-excition module^22^, hierarchical skip fusion with deep supervision^23^, or the combination of recurrent convolutional blocks with residual and attention mechanisms^24^. These developments motivate the expansion and exploration of anatomically complex regions, including the paranasal sinuses.

In parallel with advancements in CNN-based segmentation, transformer-based architectures have recently gained momentum in the medical imaging domain due to their superior capacity for global context modeling and data-driven representation learning^25^. Unlike CNNs, which are inherently limited in modeling long-range dependencies due to their local receptive fields, Transformers offer a global self-attention mechanism that enables more comprehensive integration of contextual information^25^. The introduction of the Vision Transformer (ViT) marked a shift in architectural design by applying Transformer principles directly to image data, leading to a growing number of ViT-based models in medical image analysis^26^. With their self-attention mechanism and ability to process entire images as token sequences, ViTs offer a fundamentally different approach to representation learning. This has led to their application in various medical segmentation tasks, including volumetric image segmentation^27^.While CNNs rely on a sequence of layers to capture information about the anatomical structures due to their limited receptive fields^28,29^, ViTs can achieve a significantly greater degree of freedom due to their minimal inductive bias toward input data, allowing for comprehensive integration of input data information within a single layer^29,30^. However, ViTs face an inherent limitation due to local information loss in the image-patch generation step to form a token^26,29^. To compensate, an approach based on learning the fused feature information from two models by mixing and configuring CNNs and a ViT has led to the development of hybrid networks such as TransUNet^31^, UNETR^32^, and Swin UNETR^33^. These hybrid networks capitalize on the deep and contextual understanding of images by integrating the local processing capabilities of CNNs with the long-range dependency modeling of ViTs^34–36^.

The research hypothesis of this study is as follows. CNNs, Vision Transformers (ViTs), and hybrid networks demonstrate significantly different segmentation performances in anatomically complex and morphologically variable regions of the paranasal sinuses, and among them, certain architectures can achieve an optimal trade-off between segmentation accuracy and computational efficiency suitable for clinical deployment. Therefore, the objective of this study was to compare the segmentation performance of CNNs, ViTs, and hybrid networks for the paranasal sinuses of the FS, ES, SS, and MS on CT images. Our main contributions are as follows: (1) we compared CNNs, ViTs, and hybrid networks comprehensively for the segmentation of the paranasal sinuses with differing levels of anatomical complexity and showing inflammation-induced morphological and textural changes, and (2) we also analyzed the performance of the networks in terms of segmentation accuracy and computational efficiency, demonstrating their suitability for clinical deployment in precision-guided interventions and decision support systems for sinusitis.

## Materials and methods

### Data acquisition and preparation

We included 200 patients (66 females and 134 males; mean age 49 ± 17.22 years) who were diagnosed with sinusitis (176) or normal (24) at the Gachon University Gil Medical Center (2021–2022). Patient data were obtained using a SOMATOM Definition CT scanner (Siemens Healthcare, Munich, Germany) operating at 120 kVp and 180 mAs. The CT images dimensions of 512 × 512 × 195 voxels, with voxel spacing of 0.367 × 0.367 × 0.750 mm³ and a 16-bit depth. This study was performed with the approval of the Institutional Review Board (IRB) of Gachon University Gil Medical Center (GAIRB2020-339), and in accordance with the Declaration of Helsinki. We obtained informed consent from all participants and their legal guardians, and no identifying information of participants was included in this study.

**Fig. 1:**
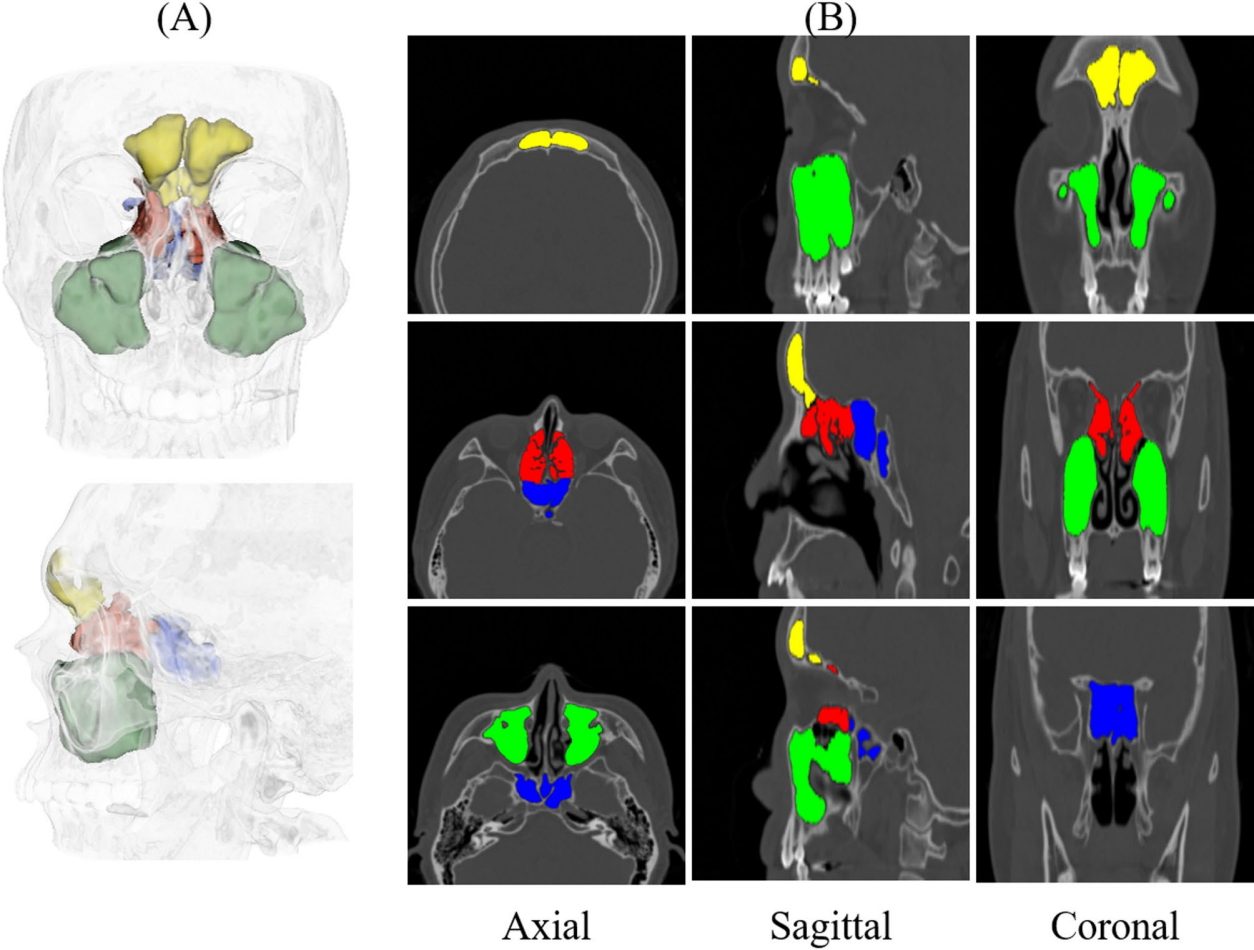
(**A**) Three-dimensional visualization of the paranasal sinuses consisting of the frontal sinus (FS), ethmoid sinus (ES), sphenoid sinus (SS), and maxillary sinus (MS) regions, represented in yellow, red, blue, and green colors, respectively. (**B**) A two-dimensional visualization of the sinuses in axial, sagittal, and coronal views with binary masks delineating each sinus in the CT image. (Created with 3D Slicer, 5.6.1, https://www.slicer.org/ ).

The ground truth annotations for the paranasal sinuses, including the frontal sinus (FS), ES, SS, and MS, were manually performed using 3D Slicer (Windows 10 version, MIT, USA) by two board-certified otorhinolaryngologists (Fig. 1)^37^. Annotations were conducted across axial, sagittal, and coronal planes, and final segmentation boundaries were determined through consensus between the two physicians to ensure anatomical consistency. In regions with complex structural overlap, such as the interface between FS and anterior ethmoid cells or the inferior boundary of the ES, bony landmarks and standard anatomical planes were used as reference. For training the deep learning models, we allocated 117 volumes to the training set and 39 to the validation set. The test set was composed exclusively of 40 volumes from patients diagnosed with sinusitis. Four volumes were excluded due to low image quality. All CT volumes were resized to 256 × 256 × 128 voxels to accommodate GPU memory limitations.

We estimated an appropriate dataset size for comparing segmentation accuracy across CNNs, ViTs, and hybrid networks through a power analysis based on a repeated-measures ANOVA. As all six networks were applied to the same set of test subjects, we assumed a within-subject design. The analysis was performed using G*Power (Version 3.1.9.4; Universität Düsseldorf, Germany), assuming a mean accuracy difference of 0.05 and a standard deviation of 0.10. Considering the potential variability in CT acquisition across patients, we considered factors such as differences in patient positioning, image noise, and contrast levels. Based on this consideration, we set the statistical parameters to a correlation of 0.3 among repeated measures, a nonsphericity correction factor of ε = 0.7, a significance level of 0.05, a statistical power of 0.80, and an effect size of 0.25. Based on these parameters, the estimated minimum required sample size was *N* = 37. Accordingly, we retained the dataset composition of 117, 39, and 40 volumes for training, validation, and testing, respectively.

### 3D convolutional neural networks for volumetric image segmentation

**Fig. 2:**
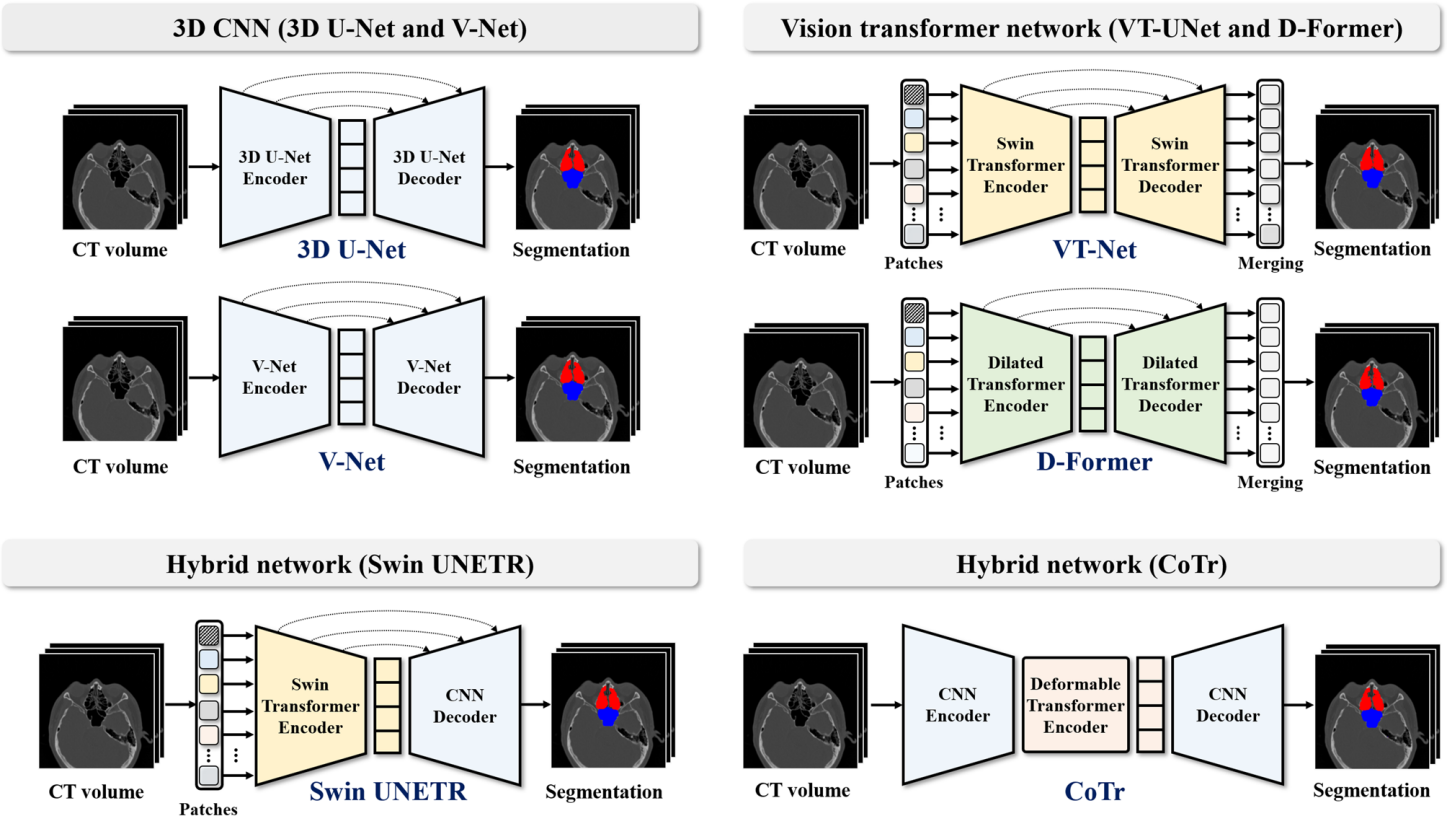
The architectures of convolutional neural networks (CNNs) of (**A**) 3D U-Net and V-Net, vision transformers (ViTs) of (**B**) VT-UNet and (**C**) D-Former, and hybrid networks of (**D**) Swin UNETR, and (**E**) CoTr.

We utilized two 3D CNNs, 3D U-Net^38^ and V-Net^39^, for volumetric image segmentation (Fig. 2). The 3D U-Net, an extension of the original 2D U-Net, incorporated 3D convolutions while maintaining the core architecture of the 2D U-Net, enabling processing of 3D images effectively^38,40^. Similarly, V-Net^39^ was designed to enhance volumetric learning through residual connections and 5 × 5 × 5 convolutional kernels in the encoder. It replaced max pooling with convolutional downsampling and maintained an overall structure similar to the 3D U-Net^39^.

### Vision transformers for volumetric image segmentation

Accordingly, Vision Transformers (ViTs) have emerged, applying the principles of transformers to overcome this limitation can be operated by dividing input images into patches, each supplemented with positional information^26^. We used the VT-UNet^41^ and D-Former^42^ ViTs for volumetric image segmentation (Fig. 2). VT-UNet adopted a hierarchical transformer architecture inspired by the Swin Transformer, using window-based and shifted-window attention mechanisms at each stage to preserve spatial locality and enhance context modeling^41,43^. The decoder mirrored the encoder’s hierarchical structure but introduced parallel cross-attention and self-attention at equivalent stages, with skip connections transferring key and value information to ensure continuity in the information flow^41^.

The D-Former distinguished itself from typical ViT architectures by employing depth-wise convolution for positional encoding, enabling dynamic acquisition of positional information while reducing computational overhead and enhancing translation invariance^42^. It also incorporated a multi-head self-attention mechanism that evaluated adjacent and distant patches to gather dense contextual information for the predicted target, allowing for efficient encoding with fewer parameters^42^.

### Hybrid networks for volumetric image segmentation

Recent advancements in image segmentation have seen the convergence of CNN and ViT architectures, each with unique strengths and limitations in processing structural and long-range dependency information^35,36^. To leverage the complementary strengths of both architectures, hybrid networks have emerged, combining CNNs and transformers to overcome their individual limitations^44^. Representative examples include TransUNet and UNETR, which integrate the strengths of CNNs and ViTs: TransUNet employs a CNN–transformer encoder followed by a CNN decoder^31^, while UNETR uses a transformer encoder with a CNN decoder^32^. We used two hybrid networks, Swin UNETR^25^ and CoTr^45^, for volumetric image segmentation (Fig. 2). Swin UNETR^33^ employed a Swin Transformer encoder within a U-shaped architecture derived from UNETR^32^, integrating window-based attention with CNN-based decoding. It included residual convolutional blocks and instance normalization at each skip-connection stage to enhance the transfer of information from the encoder from decoder^25,30^.

By comparison, CoTr^45^, building on the integration concept of TransUNet^31^, implemented a cascaded encoder structure consisting of a CNN followed by a deformable transformer^46–48^. The CNN encoder first extracted high-resolution features, which were then refined by the transformer using a sparse query-key attention mechanism focused on spatially relevant regions, thereby reducing computational and spatial complexity^45–48^. The decoder, fully CNN-based, upsampled the encoded features and employed skip connections to retain low-level detail with minimal loss. Through cascaded sequence, CoTr effectively preserved both local and global information throughout the segmentation process. Table 1 provided a comparative overview of the architectural characteristics of CNNs, ViTs, and hybrid networks, including encoder–decoder design, positional encoding strategies, attention mechanisms, skip connections, and main structural features.

**Table 1:** Architectural comparison of cnns, vision transformers, and hybrid networks, categorized by encoder and decoder designs, positional encoding and attention types, skip connections, and architectural features.

Network	Architecture	Encoder	Encoder Details	Decoder
3D U-Net	CNNs	CNN	Hierarchical features with convolution and pooling operations	Symmetric to the Encoder
V-Net	CNNs	CNN	Residual blocks with 5$\:\times\:$5$\:\times\:$5 conv	Symmetric to the Encoder
VT-UNet	ViTs	Transformer	Hierarchical window features with Swin Transformer encoder	Encoder-based decoder with parallel cross attention
D-Former	ViTs	Transformer	Transformer encoder with depthwise convolution	Symmetric to the Encoder
Swin UNETR	Hybrid	Transformer	Hierarchical window features with Swin Transformer encoder	CNN with residual blocks
CoTr	Hybrid	CNN-Transformer Cascade	Cascaded encoder structure combining CNN and deformable transformer modules	CNN

Positional Encoding	Attention Type	Skip Connections	Structural Features	
-	-	Stage-wise Skip Connections	Extension of U-Net to volumetric data	
-	-	Stage-wise Skip Connections	Equivalent to 3D U-Net with enlarged 5 × 5 × 5 kernels	
Relative Positional Encoding	Window & Shifted Window Attention	Stage-wise Skip Connections via shared key/value	Cross/Self Attention Fusion at Decoder Stages	
Depthwise Convolution-based Positional Encoding	Multi-head attention capturing local and global contexts	Stage-wise Skip Connections	Lightweight ViT with Local and Global Attention	
Relative Positional Encoding	Window & Shifted Window Attention	InstanceNorm-Enhanced Skip Connections	Swin Transformer encoder fused with CNN-based decoding	
Sparse key-based guidance Positional Encoding	CNN + Transformer	Skip connections from CNN encoder to CNN decoder	CNN-Transformer cascaded with deformable attention	

### Implementation details

To compare the segmentation performance of networks, we conducted multi-class segmentation of the background, FS, ES, SS, and MS. We adopted an equal ratio of a dice similarity coefficient (DSC) score^49,50^ and cross-entropy loss^51^ for multi-class loss functions. All networks were implemented in PyTorch and trained on a single NVIDIA RTX A6000 48GB GPU using the default hyperparameter values provided in their official GitHub repositories (https://github.com/faustomilletari/VNet, https://github.com/himashi92/vt-unet, https://github.com/kkk55596/D-Former, https://github.com/Project-MONAI/research-contributions/tree/main/SwinUNETR, and https://github.com/YtongXie/CoTr) and original papers^33,38,39,41,42,46^.

The detailed hyperparameter configurations are as follows. Both 3D U-Net and V-Net employed a four-stage encoder–decoder structure, with feature channels doubling from 32 to 256 across stages. 3D U-Net used 3 × 3 × 3 convolutional kernels and max pooling with a stride of 2 for downsampling, while V-Net adopted 5 × 5 × 5 convolutions, with strided convolutions for downsampling and transposed convolutions for upsampling. Transformer-based variants utilized architecture-specific patch embeddings and attention mechanisms. VT-UNet was configured with a patch size of (64, 192, 160) and 30 feature channels, and D-Former adopted a hierarchical Transformer architecture with a patch size of (4, 4, 4) and an embedding dimension of 96. Depths, attention heads, and group size of D-Former were set to (2, 2, 6, 2), (3, 6, 12, 24), and (2, 7, 7), respectively. In hybrid networks, convolutional and Transformer modules were integrated while maintaining comparable architectural scales. Swin UNETR used a patch size of 96, embedding dimension 192, and window size 7 × 7 × 7, with stage depths (2, 2, 6, 2) and attention heads (3, 6, 12, 24). CoTr employed a ResNet-50 encoder with 4 attention layers, each containing 8 heads and a hidden dimension of 512. The patch size for Transformer input was set to one-eighth of the input resolution. All networks were trained for 200 epochs with a batch size of 1.

During training, we applied augmentation using TorchIO, focusing on contrastive transformations including RandomBiasField, RandomGamma with a log-gamma range of (–0.3, 0.3), and RescaleIntensity to a range of (0, 1). Spatial transformations such as flipping or rotation were not applied in order to preserve the anatomical orientation of the 3D CT volumes and avoid disrupting inter-slice spatial consistency^52^.

### Performance evaluation of segmentation

To evaluate the segmentation performance for the paranasal sinuses, we used six evaluation metrics: the Jaccard Index ($\:\text{J}\text{I}=\frac{TP}{TP+FN+FP}$), Dice similarity score$\:\:(\text{D}\text{S}\text{C}=\frac{2TP}{2TP+FN+FP}$), precision ($\:\text{P}\text{R}=\frac{TP}{TP+FP}$), recall ($\:\text{R}\text{C}=\frac{TP}{TP+FN}$), and 95% Hausdorff Distance (HD95). Overlap-based evaluation metrics such as the JI, DSC, PR, and RC can be seen as metrics tailored to evaluate the areas of segmentation results. Specifically, HD95 (Eq. 1) served as a distance-based evaluation metric for evaluating segmented boundaries and provided a robust and reliable way to evaluate how well the boundaries of a segmentation result matched the boundaries of the ground truth^53,54^, which can be defined as:

1 $$\:\text{H}\text{D}95\left(\text{G},\text{P}\right)=\text{max}\left\{{\text{sup}}_{\text{g}\in\:G}d(g,P),{\text{sup}}_{\text{p}\in\:P}d(G,p)\right\}$$

In clinical settings for automatic paranasal sinus segmentation, learning outcomes, the efficiency of the learning process, and the scalability of learning across various variations are required collectively^55^. Accordingly, computational efficiency has become an increasingly important consideration for evaluating the practicality of deep learning models in real-world clinical settings^56^. To address this, we assessed the learning efficiency of each network by comparing the number of parameters (Params) and inference time (IT). The IT was measured as the average processing time per 3D volume in seconds.

We conducted repeated-measures ANOVA tests to evaluate segmentation performance differences among CNNs, ViTs, and hybrid networks. Bonferroni-corrected post hoc tests were then performed to conduct pairwise comparisons among the six networks. All statistical analyses were conducted using Python (version 3.8.16) with the SciPy (version 1.10.1), with the Statsmodels (version 0.12.2) and Pingouin (version 0.5.5), and Scikit-learn (version 1.2.2) libraries. Statistical significance was defined as 0.05.

## Results

We compared the segmentation performance and model complexity of the 3D U-Net and V-Net CNNs and VT-UNet and D-Former ViTs, and hybrid networks of Swin UNETR and CoTr for the paranasal sinuses from patients with sinusitis. Table 2 presents the mean segmentation performance metrics (JI, DSC, PR, RC, and HD95) for the networks across all paranasal sinuses. The results in Table 2 indicate that the Swin UNETR outperformed other networks in most segmentation metrics. Except PR of 0.935, Swin UNETR achieved the highest mean segmentation scores, with a JI of 0.719, a DSC of 0.830, and an RC of 0.758, and the lowest HD95 value of 10.529 for the paranasal sinuses, along with the smallest number of its architectural parameter. Notably, Swin UNETR exhibited statistically significant differences in JI, DSC, PR, and RC metrics compared to ViT-based networks (*p* < 0.05), and similar significant differences were also observed in DSC and RC metrics compared to CNN-based networks (*p* < 0.05). CoTr achieved a performance comparable to that of the Swin UNETR with a JI of 0.707, a DSC of 0.820, a PR of 0.934, an RC of 0.741, and an HD95 value of 11.618. The V-Net achieved a performance similar to that of Swin UNETR (a JI of 0.696, PR of 0.946, and HD95 value of 10.947). CoTr also showed the shortest inference time (IT) at 0.149 s per volume across networks, with the largest gap observed against V-Net, which recorded the slowest IT of 0.646 s (Table 2). However, both CoTr and V-Net had the larger number of its architectural parameter, while the VT-UNet and D-Former showed the lowest performance for all metrics (Table 2).

**Table 2:** The mean segmentation performance of the Jaccard index (JI), dice similarity coefficient (DSC), precision (PR), recall (RC), and 95% hausdorff distance (HD95) with the number of parameters (Params) for the paranasal sinuses using CNNs (3D U-Net and V-Net), vision Transformers (VT-UNet and D-Former), and hybrid networks (Swin UNETR and CoTr) (*statistically significant difference in JI from Swin UNETR (*p* < 0.05), ^†^Statistically significant difference in DSC from Swin UNETR (*p* < 0.05), and ^‡^Statistically significant difference in RC from Swin UNETR (*p* < 0.05)).

Network	JI $\:\uparrow\:$	DSC $\:\uparrow\:$	PR $\:\uparrow\:$	RC $\:\uparrow\:$	HD95 $\:\downarrow\:$	Params(M)$\:\:\downarrow\:$	IT (Sec) $\:\downarrow\:$
3D U-Net	0.692 ± 0.148	0.808 ± 0.117^†^	0.934 ± 0.085	0.728 ± 0.149	12.485 ± 10.948	35.971	0.232 ± 0.001
V-Net	0.696 ± 0.159	0.808 ± 0.140^†^	**0.946** **± 0.102**	0.722 ± 0.158^‡^	10.947 ± 11.604	45.616	0.646 ± 0.004
VT-UNet	0.633 ± 0.165*	0.761 ± 0.144^†^	0.875 ± 0.147	0.695 ± 0.161^‡^	14.336 ± 9.569	20.751	0.275 ± 0.148
D-Former	0.684 ± 0.148*	0.802 ± 0.121^†^	0.917 ± 0.109	0.726 ± 0.138^‡^	13.841 ± 13.289	50.551	0.203 ± 0.001
Swin UNETR	**0.719** **± 0.123**	**0.830** **± 0.097**	0.935 ± 0.116	**0.758** **± 0.099**	**10.529** **± 9.017**	**15.705**	0.376 ± 0.001
CoTr	0.707 ± 0.139	0.820 ± 0.110	0.934 ± 0.107	0.741 ± 0.129	11.618 ± 12.005	41.866	**0.149** ** ± 0.002**

Table 3 provides detailed performance metrics (JI, DSC, PR, RC, and HD95) for each individual paranasal sinus of the FS, ES, SS, and MS with sinusitis. The results in Table 3 indicate that all networks achieved the best segmentation performance in the MS, which has the least anatomical complexity of the paranasal sinuses; the lowest performance in the ES, which has the highest anatomical complexity; and moderate performance in the FS and SS, which exhibit intermediate anatomical complexity (Table 3). The Swin UNETR achieved the highest segmentation accuracies of 0.710 and 0.647 for the JI and 0.825 and 0.783 for the DSC in the FS and ES, respectively, indicating a relatively high degree of anatomical complexity of the paranasal sinuses. Accordingly, Swin UNETR showed statistically significant differences in all metrics except PR in the FS region (*p* < 0.05). Specifically, significant differences in JI and HD95 were found compared to both CNNs and the ViTs (*p* < 0.05). For DSC, significant differences were observed compared to CNNs and VT-UNet, and for RC, compared to all networks except CoTr (*p* < 0.05). In the ES region, Swin UNETR exhibited significant differences in JI, DSC, and RC across all networks (*p* < 0.05). However, the comparable performance of 0.726 and 0.794 of the JI and 0.832 and 0.880 of the DSC in the SS and MS, respectively, with 3D U-Net’s 0.727 and 0.799 JI and 0.835 and 0.885 DSC in the SS and MS, respectively, indicating a relatively low anatomical complexity (Table 3). When evaluating segmentation boundaries, the Swin UNETR achieved the lowest error of the HD95 of 10.552 in the FS, and the CoTr the HD95 of 6.906 in the MS, while V-Net achieved the lowest HD95 of 10.954 and 10.409 in the ES and SS, respectively. Based on Table 3, A statistically significant difference in HD95 was observed between Swin UNETR and CNNs and VT-UNet in the FS (*p* < 0.05), and CoTr exhibiting a significant difference compared to the ViTs in the MS (*p* < 0.05).

**Table 3:** Segmentation performance of the Jaccard index (JI), dice similarity coefficient (DSC), precision (PR), recall (RC), and 95% hausdorff distance (HD95) for the frontal sinus (FS), ethmoid sinus (ES), sphenoid sinus (SS), and maxillary sinus (MS) using CNNs (3D U-Net and V-Net), vision Transformers (VT-UNet and D-Former), and hybrid networks (Swin UNETR and CoTr) (*significant difference in all metrics except PR in Swin UNETR in FS (p-value < 0.05), ^†^Significant difference in JI, DSC, RC in Swin UNETR in ES (p-value < 0.05), ^‡^Significant difference in HD95 in CoTr in MS (p-value < 0.05)).

Network	JI $\:\uparrow\:$				DSC $\:\uparrow\:$						
	FS	ES	SS	MS	FS	ES	SS	MS			
3D U-Net	0.660 ± 0.158*	0.582 ± 0.106^†^	**0.727** ** ± 0.126**	0.799 ± 0.096	0.782 ± 0.138*	0.730 ± 0.092^†^	**0.835** ** ± 0.095**	0.885 ± 0.069			
V-Net	0.679 ± 0.156*	0.627 ± 0.101	0.699 ± 0.196	0.779 ± 0.129	0.797 ± 0.132	0.765 ± 0.090	0.802 ± 0.189	0.868 ± 0.107			
VT-UNet	0.589 ± 0.172*	0.575 ± 0.100^†^	0.618 ± 0.197	0.748 ± 0.110*	0.725 ± 0.160*	0.725 ± 0.083^†^	0.743 ± 0.179	0.850 ± 0.084			
D-Former	0.664 ± 0.154*	0.604 ± 0.087^†^	0.683 ± 0.173	0.786 ± 0.098	0.786 ± 0.132*	0.749 ± 0.071^†^	0.797 ± 0.144	0.876 ± 0.080			
Swin UNETR	**0.710** ** ± 0.110**	**0.647** ** ± 0.075**	0.726 ± 0.142	0.794 ± 0.106	**0.825** ** ± 0.086**	**0.783** ** ± 0.057**	0.832 ± 0.117	0.880 ± 0.091			
CoTr	0.695 ± 0.123	0.612 ± 0.085^†^	0.712 ± 0.161	**0.810** ** ± 0.095**	0.813 ± 0.095	0.756 ± 0.068^†^	0.819 ± 0.143*	**0.892** ** ± 0.073**			

PR $\:\uparrow\:$				RC $\:\uparrow\:$				HD95 $\:\downarrow\:$			
FS	ES	SS	MS	FS	ES	SS	MS	FS	ES	SS	MS
0.930 ± 0.112	0.910 ± 0.069	0.933 ± 0.074	0.962 ± 0.066	0.696 ± 0.156*	0.623 ± 0.126^†^	0.772 ± 0.135	0.822 ± 0.086	14.497 ± 12.135*	12.953 ± 4.948	12.862 ± 11.735	9.629 ± 12.547
0.942 ± 0.101	**0.920** ** ± 0.062**	**0.951** ** ± 0.129**	**0.971** ** ± 0.099**	0.709 ± 0.152*	0.669 ± 0.118	0.717 ± 0.203	0.792 ± 0.116	14.818 ± 18.902*	**10.954** ** ± 4.440**	**10.409** ** ± 9.672**	7.606 ± 6.455
0.928 ± 0.137	0.820 ± 0.105	0.844 ± 0.195	0.907 ± 0.104	0.614 ± 0.173*	0.665 ± 0.112^†^	0.696 ± 0.186	0.805 ± 0.083	13.414 ± 10.113*	12.737 ± 4.874	17.484 ± 11.799	13.708 ± 9.345^‡^
0.929 ± 0.123	0.895 ± 0.063	0.907 ± 0.126	0.938 ± 0.108	0.701 ± 0.140*	0.652 ± 0.098^†^	0.725 ± 0.166	0.826 ± 0.063	11.309 ± 9.991	11.295 ± 4.422	16.492 ± 18.154	16.267 ± 15.219^‡^
**0.943** ** ± 0.117**	0.902 ± 0.072	0.928 ± 0.141	0.965 ± 0.113	**0.746** ** ± 0.088**	**0.699** ** ± 0.083**	**0.772** ** ± 0.109**	0.812 ± 0.078	**10.552** ** ± 8.627**	11.171 ± 5.249	13.124 ± 12.944	7.269 ± 6.159
0.935 ± 0.104	0.902 ± 0.068	0.938 ± 0.142	0.962 ± 0.093	0.732 ± 0.112	0.660 ± 0.099^†^	0.740 ± 0.157	**0.834** ** ± 0.066**	13.225 ± 13.540	11.421 ± 3.933	14.922 ± 17.459	**6.906** ** ± 6.095**

**Fig. 3:**
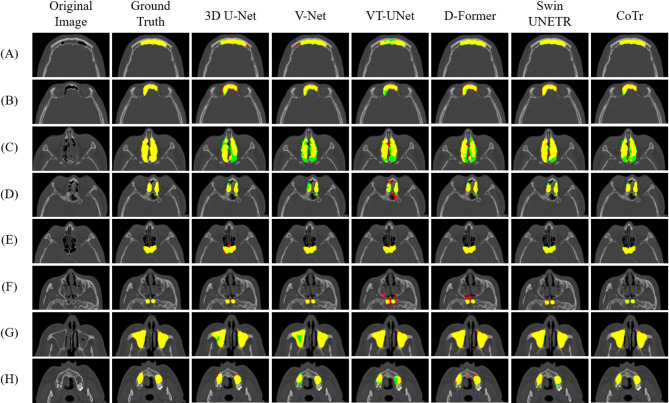
Segmentation results of the paranasal sinuses by CNNs (3D U-Net and V-Net), ViTs (VT-UNet and D-Former), and hybrid networks (Swin UNETR and CoTr) for patients with sinusitis. The rows from top to bottom represent the frontal sinus (**A**) and (**B**), ethmoid sinus (**C**) and (**D**), sphenoid sinus (**E**) and (**F**), and maxillary sinus (**G**) and (**H**). True positives, false negatives, and false positives by segmentation result are in yellow, green, and red, respectively.

**Fig. 4:**
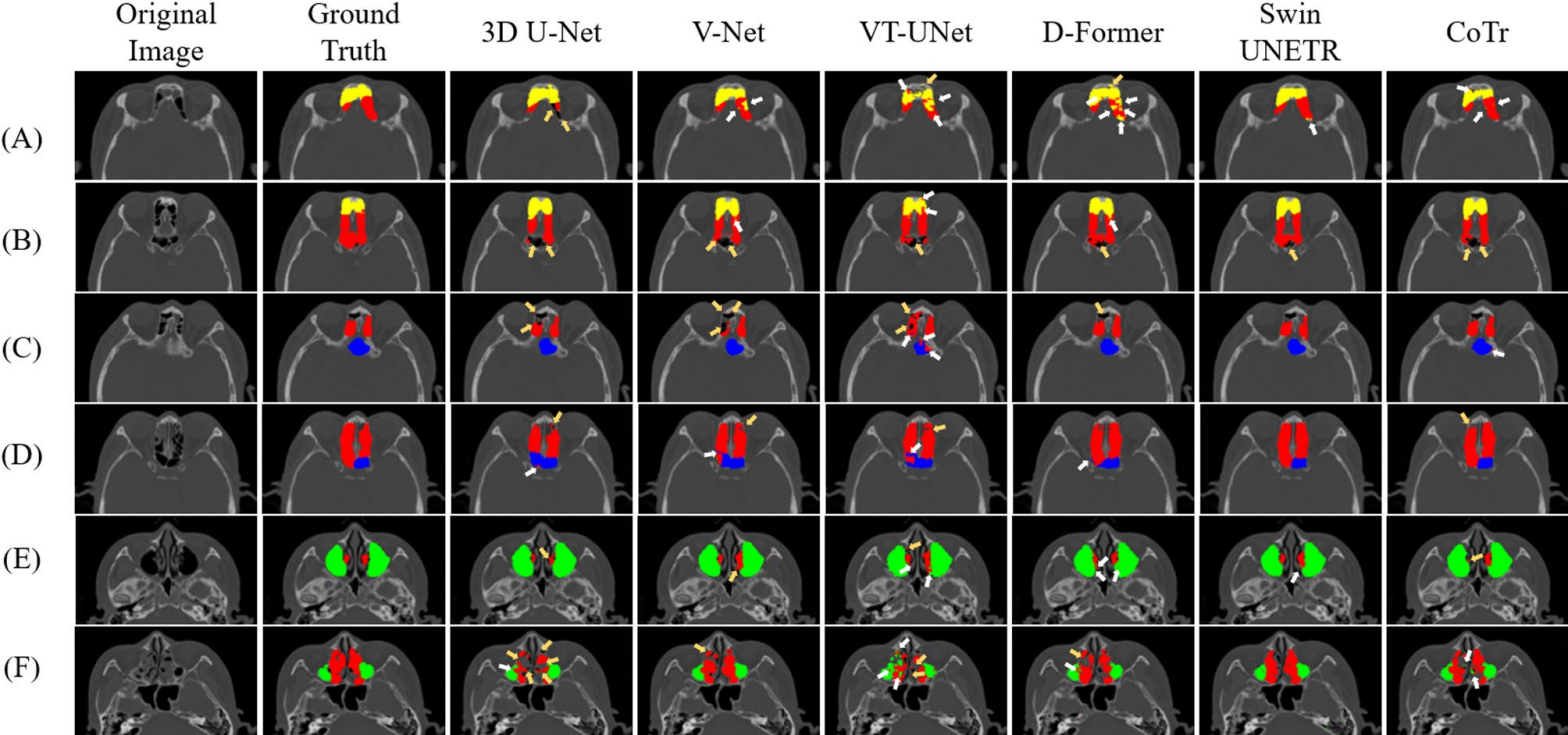
A 2D visualization of segmentation results for the frontal sinus (yellow), ethmoid sinus (red), sphenoid sinus (blue), and maxillary sinus (green) with a focus on boundary areas for the frontal sinus and ethmoid sinus (**A** and **B**), ethmoid sinus and sphenoid sinus (**C** and **D**), and ethmoid sinus and maxillary sinus (*E* and **F**) for patients with sinusitis. White and brown arrows indicate failures to accurately delineate boundaries between sinuses, and between sinuses and adjacent structures, respectively.

For qualitative evaluation, we visualized the segmentation results of the paranasal sinuses using CNNs, ViTs, and hybrid networks for patients with sinusitis (Fig. 3). The hybrid networks produced fewer false positives and false negatives overall compared with the CNNs and ViTs for sinuses with morphological and textural variations by sinusitis. Specifically, CNNs showed more false negatives in the FS and ES, resulting in a lower RC compared with the hybrid networks, while ViTs produced more false positives in the SS and MS, resulting in a lower PR than the hybrid (Fig. 3; Table 2). We also emphasized the segmentation boundaries in the visualizations, particularly between the FS and ES, the ES and SS, and the ES and MS for patients with sinusitis (Fig. 4). The hybrid networks most accurately delineated these boundaries, closely matching the ground truth (Fig. 4). In contrast, CNNs showed more confusion in segmenting sinus areas, with one region encroaching into another (white arrow), and ViTs more often failing to clearly distinguish the boundaries between paranasal sinuses (white arrow), and between paranasal sinuses and adjacent structures (brown arrow) (Fig. 4). These segmentation errors by CNNs and ViTs were more pronounced between the FS and ES. More segmentation errors were made by ViTs in distinguishing between ES and the nasal cavity, with over-segmentation causing ES to extend beyond its boundary (Fig. 4).

**Fig. 5:**
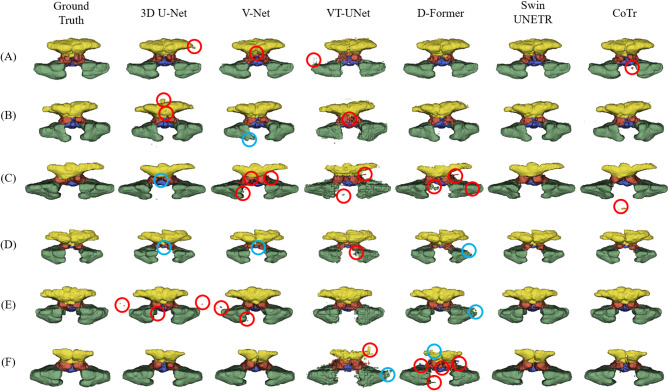
A three-dimensional visualization of the frontal sinus (yellow), ethmoid sinus (red), sphenoid sinus (blue), and maxillary sinus (green) from ground truth and segmentation results by 3D U-Net, V-Net, VT-UNet, D-Former, Swin UNETR, and CoTr for patients with sinusitis (A-F). Red and blue circles represent false positives and false negatives, respectively. (Created with 3D Slicer, 5.6.1, https://www.slicer.org/ ).

**Fig. 6:**
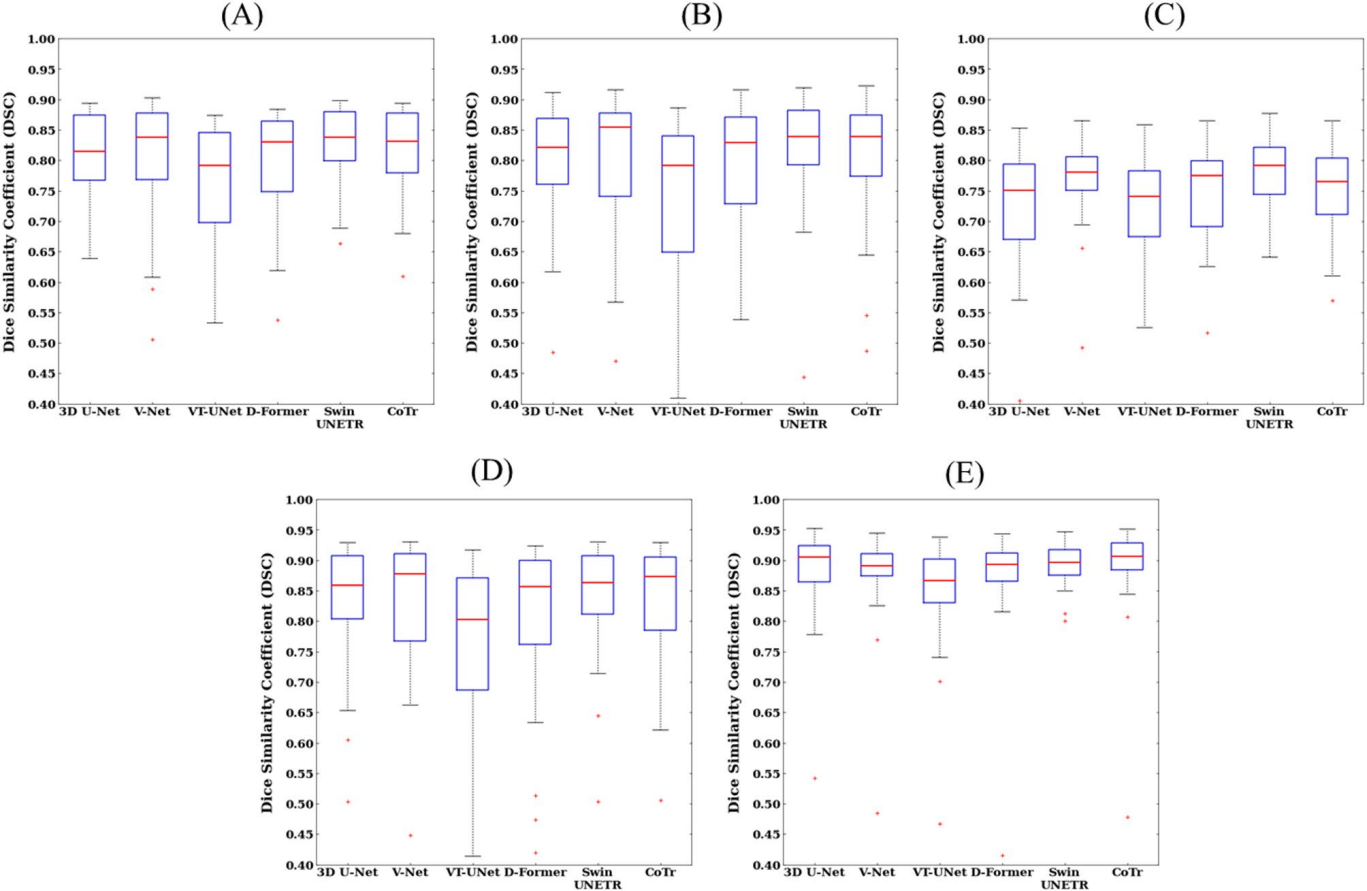
Boxplots of the Dice Similarity Coefficient (DSC) values for 3D U-Net, V-Net, VT-UNet, D-Former, Swin UNETR, and CoTr. From top to bottom, each row denotes the values for (**A**) average performance, (**B**) frontal sinus, (**C**) ethmoid sinus, (**D**) sphenoid sinus, and (**E**) maxillary sinus. Each boxplot contains the first and third quartiles of data, with medians located inside the boxes and visualized as red lines. The whiskers extend above and below each box by 1.5 times the interquartile range (IQR), and outliers are visualized as red plus marks indicating values 1.5 IQR away from the box. A significant difference between Swin UNETR and 3D U-Net, V-Net, VT-UNet and D-Former in (**A**) (*p* < 0.05). A significant difference between Swin UNETR and 3D U-Net, VT-UNet and D-Former in (**B**) (*p* < 0.05). A significant difference between Swin UNETR and 3D U-Net, VT-UNet, D-Former and CoTr in (**C**) (*p* < 0.05).

**Fig. 7:**
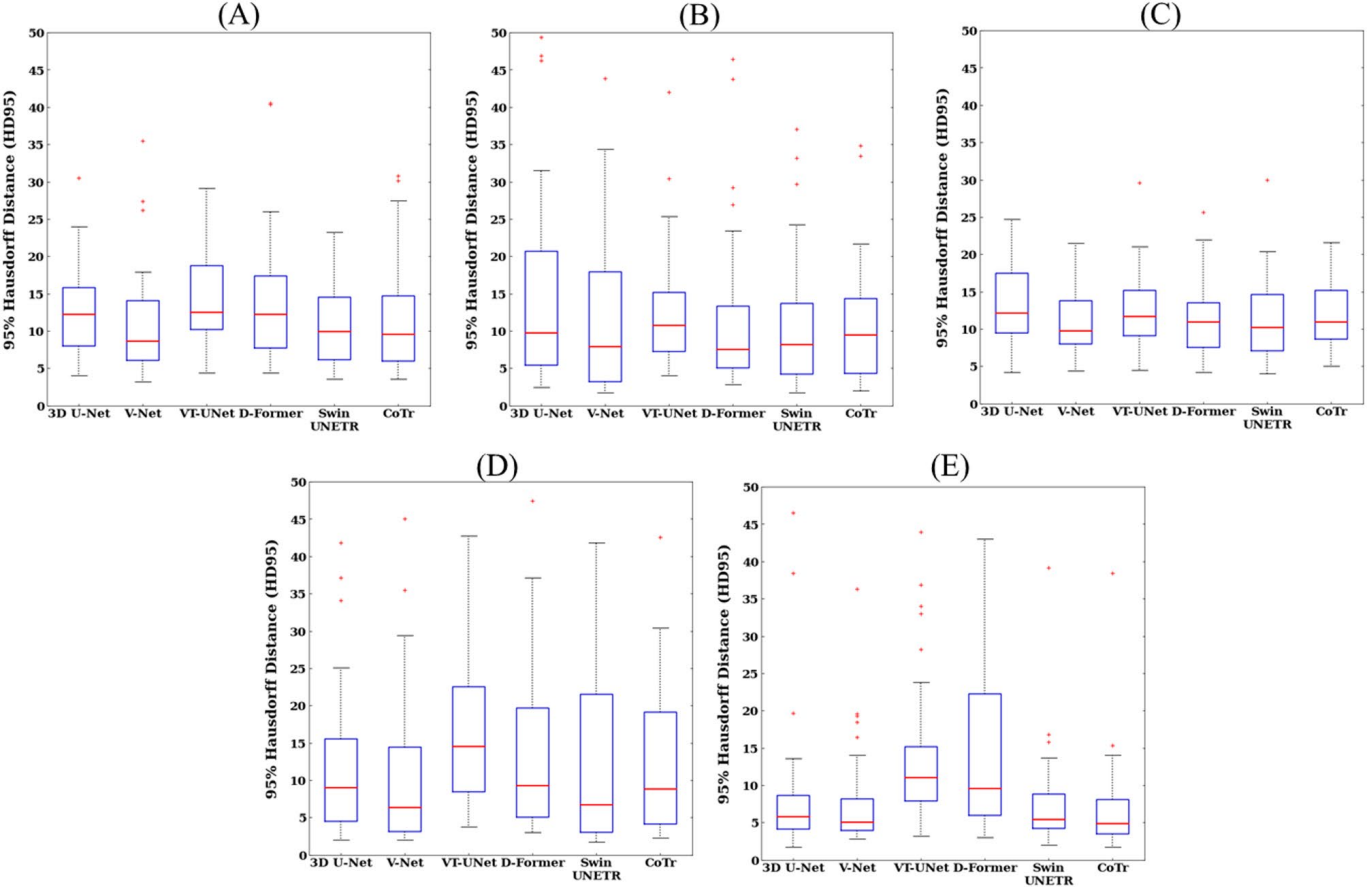
Boxplots of the 95% Hausdorff Distance (HD95) values for 3D U-Net, V-Net, VT-UNet, D-Former, Swin UNETR, and CoTr. From top to bottom, each row denotes the values for (**A**) average performance, (**B**) frontal sinus, (**C**) ethmoid sinus, (**D**) sphenoid sinus, and (**E**) maxillary sinus. Each boxplot contains the first and third quartiles of data, with medians located inside the boxes and visualized as red lines. The whiskers extend above and below each box by 1.5 times the interquartile range (IQR), and outliers are visualized as red plus marks indicating values 1.5 IQR away from the box. A significant difference between Swin UNETR and 3D U-Net, V-Net, and VT-UNet in (**B**) (*p* < 0.05). A significant difference between CoTr and VT-UNet and D-Former in (**E**) (*p* < 0.05).

The 3D visualization of predictions for the four paranasal sinuses show that the volume predicted by the Swin UNETR has fewer false positive (red circle) and fewer false negative (blue circle) volumes compared with the CNNs and ViTs (Fig. 5). The CNNs of 3D U-Net and V-Net misidentified some global relational characteristics of the repetitive structures leading to more false negatives in the FS and ES, while the ViTs of VT-UNet and D-Former tended to fail to capture the local continuity leading to more false positives in the FS, ES, SS, and MS. Therefore, the Swin UNETR provided superior 3D segmentation results, showing the lowest false-positive and false-negative volumes across all sinuses. With respect to overall performance, hybrid networks demonstrated more balanced and robust performance compared to CNNs and ViTs. Figures 6 and 7 illustrate the segmentation performance distributions based on DSC (Fig. 6) and HD95 (Fig. 7), respectively. In both metrics, Swin UNETR showed consistently higher median values with smaller interquartile ranges, shorter whiskers, and fewer outliers across most sinus regions (Figs. 6 and 7). CoTr similarly exhibited a trend of reduced HD95 variability, especially in the MS region (Fig. 7).

**Fig. 8:**
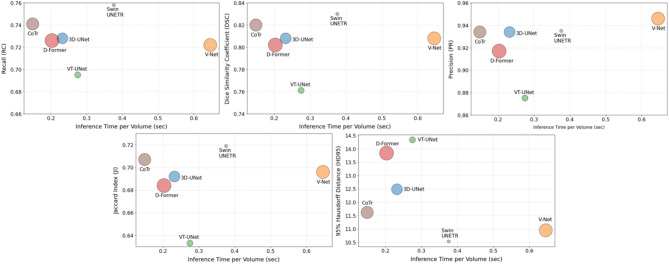
Bubble charts visualizing the trade-off between inference time and segmentation accuracy metrics for each network, including 3D U-Net, V-Net, VT-UNet, D-Former, Swin UNETR, and CoTr. The x-axis indicates the average inference time per volume, and the y-axis denotes each performance metric: (**A**) Jaccard Index (JI), (**B**) Dice Similarity Coefficient (DSC), (**C**) Precision (PR), (**D**) Recall (RC), and (**E**) 95% Hausdorff Distance (HD95). All values represent average performance across all sinuses, and the bubble size reflects the number of trainable parameters in each model.

Figure 8 provides a bubble chart overview of the trade-offs between segmentation performance, inference time per volume, and the number of parameters across all networks. Among the evaluated models, CoTr exhibited the most balanced performance tendency across segmentation accuracy, inference time, and parameter count. It consistently showed above-average segmentation performance, with the fastest inference time among all models and a mid-sized parameter count (41.9 M). Notably, V-Net showed comparable segmentation accuracy to 3D U-Net but exhibited the longest inference time among all models, indicating a clear disadvantage in computational efficiency. ViTs and 3D U-Net demonstrated inference times between those of CoTr and Swin UNETR. However, they consistently exhibited lower segmentation performance than the hybrid models. Swin UNETR exhibited a longer inference time compared to other models, but simultaneously demonstrated the highest segmentation accuracy with the lowest number of parameters (15.7 M) (Table 2; Fig. 8).

In conclusion, the segmentation performance and results indicated that hybrid networks combining CNNs and ViTs demonstrated the most accurate segmentation of the paranasal sinuses, effectively learning the anatomical complexity as well as the morphological and textural variations related to sinusitis, compared with other networks for patients with sinusitis.

## Discussion

Chronic rhinosinusitis (CRS) often leads to complex anatomical and pathological variations in the paranasal sinuses, including mucosal thickening, bony remodeling, and obscured sinus boundaries^2,3^. These variations pose significant challenges for accurate CT-based segmentation, which is valuable for applications such as image-guided surgery and 3D volumetric staging^6,9,12–14^. Although previous studies have primarily explored CNN-based models for the automatic segmentation of the paranasal sinuses^16–20^, to our knowledge, no prior study has conducted a systematic comparison of CNNs, ViTs, and hybrid networks across sinuses with varying anatomical complexity. To address this research gap, we comparatively evaluated six representative architectures including two CNNs, two ViTs, and two hybrid networks for automatic segmentation of the paranasal sinuses.

Considering the anatomical complexity of the paranasal sinuses and their interwoven relationships with surrounding structures^57^, segmentation errors from CNNs and ViTs were frequently observed in regions adjacent to surgical landmarks that are critical for maintaining anatomical validity in ESS planning. CNNs tended to under-segment the FS and ES (Fig. 3), particularly near transition zones such as the ethmoido-frontal junction, where anatomical boundaries may be ambiguous due to interleaved ethmoidal air cells or agger nasi variants^58^. These regions are often delineated in clinical practice using landmarks such as the ground lamella or fovea ethmoidalis^58^, and the resulting boundary ambiguity was qualitatively illustrated in Fig. 4, where CNN predictions blurred the FS–ES boundaries. In contrast, ViTs produced more false positives in the SS and MS regions (Fig. 3), often extending beyond sinus boundaries into adjacent non-sinus structures. These oversegmentations frequently occurred near anatomical landmarks such as the posterior ethmoid wall or orbital floor, which serve as key reference points for segmenting the ES, SS, and MS regions^58^. Additionally, oversegmentation observed in the anterior FS and ES (Figs. 3 and 4) was frequently located near regions anatomically adjacent to the agger nasi cells, a key anatomical site involved in frontal recess drainage and often implicated in early sinonasal obstruction or inflammation^58^. In comparison, hybrid networks showed fewer segmentation errors in these clinically sensitive regions, suggesting improved robustness in preserving anatomical boundaries critical for safe surgical planning. Their performance more reflected anatomical complexity and spatial continuity, both of which are essential for the reliability of automated assistance during ESS^57,58^. Collectively, segmentation errors from CNNs and ViTs tended to occur near surgically relevant regions, whereas hybrid networks demonstrated superior boundary preservation and robustness in anatomically complex areas.

The ViT-based models were not always superior to CNNs in paranasal sinus segmentation, likely due to two main factors. First, the VT-UNet and D-Former ViTs captured some global context but struggled with finer anatomical details, particularly in the FS and ES, which have relatively high anatomical complexity of the paranasal sinuses. They also had difficulty maintaining local continuity in the FS and ES, which led to more segmentation errors, this is likely due to ViTs’ lack of inductive biases, such as locality and translation invariance, limiting their ability to accurately capture smaller, detailed structures^25^. Our relatively small dataset may have contributed to these results, as ViTs generally need a larger dataset to effectively learn global and local patterns. With a limited dataset, the ViT models may have struggled to converge, leading to overfitting or missing finer anatomical details^25,30,59^. In ViT models, the lack of inductive biases make convergence difficult in small datasets resulting in the loss of 3D information, challenging the learning of representations for the paranasal sinuses of anatomical complexity and variations related to sinusitis in our study^25,59,60^.

The 3D CNNs in our study demonstrated segmentation tendencies consistent with those reported in previous studies on CNN-based sinus segmentation. 3D CNNs have some inherent limitations, as highlighted in previous studies^16–20^. Although architectures like 3D U-Net and V-Net have shown effective performance, their restricted locality causes a loss of global context, particularly in anatomically complex regions such as the FS and ES. Kuo et al. observed that CNN-based models struggle to capture anatomical variability, particularly in closely packed structures^16^. Whangbo et al. also reported that CNN-based models exhibited incomplete boundary delineation in morphologically complex or inflamed sinus regions such as the frontal and ethmoid sinuses^20^. Our results confirm these findings. Specifically, CNNs misidentified structures in the FS and failed to fully capture elongated patterns in the ES, leading to segmentation errors of more false negatives. These errors were also observed in the MS, where CNNs showed incomplete segmentation of the superior and lateral regions of the MS. These failures indicate CNNs’ continued difficulty in fully interpreting the complex anatomical relationships within the paranasal sinuses of morphological and textural variations related to sinusitis.

Hybrid networks demonstrated a balanced trade-off between segmentation accuracy and computational efficiency compared to CNNs and ViTs. Among them, Swin UNETR achieved the highest segmentation accuracy across all metrics with the smallest number of parameters (15.7 M), suggesting its potential for deployment in resource-constrained clinical settings. The number of parameters, as an indicator of model size, is a critical factor for clinical deployment, particularly in relation to flexibility and scalability across low-resource environments and heterogeneous surgical settings^61,62^. In this context, Swin UNETR not only achieved superior segmentation performance, but also demonstrated deployment advantages due to its compact architecture. The combination of a U-shaped framework within a Swin transformer encoder allowed Swin UNETR to efficiently capture complex anatomical relationships in the paranasal sinuses with fewer architectural parameters. Swin UNETR also reduces the need to process an entire volume at once by focusing computational resources within localized windows^33,43^. These design characteristics support hierarchical feature extraction and multi-scale feature fusion, preserving segmentation accuracy without increasing computational loads^43,59^.

In contrast, CoTr achieved comparable segmentation accuracy to CNNs and ViTs but demonstrated the fastest inference time (0.149 s/volume) and a moderate parameter count among all models (Table 2; Fig. 8). Considering the increasing demand for real-time decision-making tools in surgical environments, rapid computation times are a critical factor for clinical feasibility^62,63^. In particular, therapeutic decisions often need to be made within minutes^63^, and in real-time surgeries that require both immediacy and spatial–temporal coherence, even latencies within seconds may pose procedural risks, depending on task complexity, anatomical constraints, and intraoperative environmental conditions^63–65^. In terms of CoTr, by restricting global attention to a subset of spatially meaningful tokens, CoTr significantly reduces redundant computations and memory overhead, thereby accelerating inference speed without sacrificing segmentation accuracy^46,48^. This architectural design explains how CoTr achieves a unique balance between structural simplicity and contextual richness, a trade-off essential for real-time clinical integration. Taken together, these results suggest that hybrid networks may offer greater clinical applicability than standalone CNNs or ViTs by balancing segmentation performance with architectural efficiency.

This study had several limitations This study had several limitations. First, we used an internal dataset from a single institution with a limited number of dataset. Although the study was designed with statistical validity, the complexity and high-dimensional variability involved in volumetric segmentation may not be comprehensively reflected by a dataset from a single institution. This uncertainty is particularly relevant in the presence of anatomical variation due to disease severity and inter-individual differences across a broader patient population. Additionally, the use of a limited dataset from a single institution may restrict the generalizability of the segmentation networks. Therefore, external validation of CNNs, ViTs, and hybrid networks on larger and more diverse datasets from multiple organizations is essential to complement the current experimental setting. Second, all networks were trained using default hyperparameter values as provided by their original papers and their official GitHub repositories^33,38,39,41,42,45^. However, fine-tuning task-specific hyperparameters for each network is crucial to achieving optimal segmentation performance for paranasal sinuses in terms of CT volumes for fair comparison^27^. Lastly, based on the above limitations, validation of the real-world applicability of automatic segmentation using CNNs, ViTs, and hybrid networks is warranted. This includes evaluating their potential integration into preoperative planning systems or automated reporting tools in otolaryngology practice. Future studies building on these directions may bridge the gap between experimental model performance and clinical applicability through real-world validation and integration.

In conclusion, among CNNs, ViTs, and hybrid networks, the hybrid models showed the most consistent segmentation across anatomically complex sinuses and achieved the best tradeoff between accuracy and computational efficiency, supporting their potential for clinical deployment.

## Conclusions

In this study, we compared the segmentation performance and model complexity of CNNs, ViTs, and hybrid networks for the paranasal sinuses, comprising the FS, ES, SS, and MS, which exhibit varying anatomical complexity and sinusitis-related variation on CT images. Among the hybrid networks, Swin UNETR demonstrated the highest segmentation accuracy with minimal architectural parameters, reflecting its strength in performance and efficiency. CoTr achieved faster inference speed and better accuracy than CNNs and ViTs, suggesting its potential utility in time-sensitive applications. Additionally, hybrid networks more accurately delineated anatomical boundaries across sinus transition zones and adjacent structures, which are closely associated with surgical landmarks. By integrating both local and global contextual features, hybrid networks achieved a favorable balance between segmentation accuracy and computational efficiency, indicating their potential utility in image-guided surgery and preoperative planning.

## Data Availability

The datasets generated and analyzed during the current study are not publicly available due to restrictions set by the Institutional Review Board of the Gachon University Gil Medical Center to protect patients privacy but are available from the corresponding author on reasonable request.
